# The role of social media in shaping public perceptions of bariatric surgery: a comprehensive analysis of Douyin data

**DOI:** 10.3389/fpubh.2026.1879955

**Published:** 2026-07-06

**Authors:** Rui Yang, Jianli Han

**Affiliations:** 1 Third Hospital of Shanxi Medical University, Shanxi Bethune Hospital, Shanxi Academy of Medical Sciences, Tongji Shanxi Hospital, Taiyuan, China; 2 General Surgery Department, Shanxi Bethune Hospital, Shanxi Academy of Medical Sciences, Third Hospital of Shanxi Medical University, Tongji Shanxi Hospital, Taiyuan, China

**Keywords:** bariatric surgery, Douyin, gender differences, public perception, regional differences, social media

## Abstract

**Objective:**

The aim of this study was to investigate the role of Douyin, a major Chinese social media platform, in shaping public perceptions of and attitudes toward bariatric surgery. It also analyzes the sex and regional variations in public involvement in the content of bariatric surgery and identifies strategies to increase accurate and efficient dissemination.

**Methods:**

Four commonly used Douyin data analysis tools were employed, including the Ocean Engine’s Trend Insight, Huitun Data, Feigua Douyin, and Xindou websites. The study analyzed four keywords: “bariatric surgery,” “surgery for weight loss,” “sleeve gastrectomy,” and “metabolic surgery.” Data collection spanned from October 4, 2024, to April 1, 2025, covering indicators such as the keyword search index, content dissemination, and user engagement. Additionally, the characteristics of content consumers, including sex, age, region, and interests were examined.

**Results:**

The search indices for the keywords “bariatric surgery” and “metabolic surgery” showed increases of 95.02 and 97.95%, respectively. However, the scores for content and dissemination showed a decreasing trend, indicating a gap between the availability of high-quality information and public demand. Sex differences were observed, with women accounting for 60% of the content consumers and 136 TGIs (target group index) compared with 72 for men. Differences between regions were also apparent, with greater participation in first-tier and new first-tier cities.

**Conclusion:**

Douyin plays an important role in shaping public perceptions of bariatric surgery. However, the current digital landscape reveals a mismatch between public interest and the dissemination of high-engagement content.

## 1 Introduction

Bariatric surgery is a surgical procedure that involves changing the structure of the digestive tract to help people with obesity lose weight and improve their metabolic status. Development has progressed from open surgery to minimally invasive surgery and then to the modern centrifugal technique (1). The number of bariatric surgeries performed worldwide is increasing every year, with 502,150 procedures performed in 24 countries and 2 regional registries in 2022 (2). In China, the introduction of foreign bariatric surgery techniques started in the late 20th century and early 21st century. However, given the relatively low number of people with obesity and the limited social acceptance of weight loss surgery at that time, the number of surgeries was low and was mainly concentrated in a few large medical facilities. As the problem of obesity has become more serious and the effectiveness of bariatric surgery has become more recognized, the number of bariatric procedures in China has gradually increased (3). By 2023, the total number of bariatric surgeries in China was approximately 37,249, with approximately 850 hospitals performing such surgeries, and 1,250 bariatric surgeons and 600 bariatric case managers, all at all-time highs (4). The growing social awareness and acceptance of bariatric surgery in China is linked to the extensive promotion of the effects of weight loss surgery on various online platforms, with Douyin^1^ being one of the most important platforms for dissemination.

In the context of infodemiology—the science of distribution and determinants of information in an electronic medium—social media platforms like Douyin have emerged as crucial channels for digital health communication (5). The high engagement rates on Douyin provide a unique opportunity for doctors, medical institutions, and public health advocates to disseminate short videos covering the principles, indications, and risks of bariatric surgery. Historically, misconceptions surrounding bariatric surgery framed it purely as a cosmetic weight-loss tool. However, the dissemination of medical knowledge on platforms like Douyin is associated with a broader public understanding of its metabolic benefits, such as its efficacy in managing diabetes, hypertension, and polycystic ovarian syndrome. Furthermore, Douyin facilitates direct doctor-patient interaction via live streaming and allows patients to share their postoperative experiences. This digital ecosystem not only increases public exposure to bariatric surgery but may also guide patients toward seeking care at standardized medical facilities. However, analyzing this dynamic digital landscape requires robust metrics to understand whether the information supply meets the public’s actual health literacy needs, a core objective of contemporary digital public health.

There are differences in the level of economic development of different regions in China, which contribute to differences in the implementation of bariatric surgery between developed and less developed regions. In less developed areas, patients may be less likely to undergo weight loss surgery because of limited access to information and poorer economic circumstances. In Chinese culture, some people still subscribe to the traditional view that “obesity is a symbol of health and wealth,” which is partly why widespread support for bariatric surgery is still lacking. Chinese adults are 34.3% overweight and 16.4% with obesity (6). However, the number of bariatric surgeries performed annually in China is far below the actual demand of the people with obesity. This paper analyzes the reasons behind the promotion of the Douyin platform, with the aim of ensuring more precise publicization of bariatric surgery in the future, strengthening its promotion and use in less developed regions and improving its accessibility. This study is grounded in the framework of infodemiology—the science of distribution and determinants of information in an electronic medium—which is crucial for understanding public health trends in the digital era (7).

## 2 Materials and methods

### 2.1 Ocean engine’s trend insight website

The Ocean Engine’s Trend Insight website^2^ is the brand for content consumption trends, with more than 30 million users on the platform and over 10 million keywords. Using scenarios for content consumption, such as Toutiao, Douyin and Watermelon Video, and supported by the data and technology assets of Ocean Engine provides insights and perspectives into content trends, industry research and marketing strategies. It also offers tools for data analysis, such as an arithmetic index and trend lists, to meet the data analytics needs of brands, marketers and content creators. The “Arithmetic Index” module (see text footnote 2) is a powerful tool for tracking the popularity of Douyin content and tracking trends, and includes more than 300 million videos, 5 million topics, and more than 3.2 million internet influencers.

### 2.2 Huitun data website

The Huitun Data website^3^ is a practical tool for analyzing live streaming data and offers functions such as analyzing the conversion of the host, the interaction with the fans and the profile of the fans. It also provides various e-commerce rankings related to live streaming, such as ranking of the show’s hosts, ranking of best sellers, and ranking of the MCN (multichannel network). It is a cloud-based data analytics platform that visualizes live streaming data and provides accurate, reliable and effective data analytics services to live streaming platforms.

### 2.3 Feigua data website

The Feigua Data website^4^ includes data from several platforms, including the WeChat public platform, the WeChat video account, Weibo, Douyin, Rednote and Bilibili. Through big data mining, machine learning and natural language processing, a large amount of data, including fan portraits, articles, videos and live streams, is analyzed from accounts. Together with strong digital marketing services, it provides users with products, technical services and industrial solutions. Feigua Douyin^5^ is a tool for analyzing and streaming big data from the Douyin e-commerce platform and focuses on the Douyin e-commerce theme. It offers services such as e-commerce trend prediction, analysis of brands and competitors, and analysis of influencers.

### 2.4 Xindou website

Xindou^6^ is a fully fledged artificial intelligence data-reporting tool for Douyin in the Newrank^7^ domain. It provides multidimensional data such as account searches, live streaming sales, popular content, best-selling products and brand marketing, helping clients monetize their account operations and place their brands in the right place.

### 2.5 Data analysis methods

Four keywords related to bariatric surgery, namely “bariatric surgery,” “surgery for weight loss,” “sleeve gastrectomy,” and “metabolic surgery,” were selected as the objects of analysis because they represent both the standard medical terminology and the most common colloquial search phrases used by the Chinese public. The data indicators of these keywords on the Douyin platform were statistically analyzed via the above four data analysis tools. These indicators include: Keyword search index: This reflects the popularity of the keyword in the domain. A higher value indicates a greater interest in the user searching for the keyword. Keyword comprehensive index: This reflects the overall popularity of the keyword on Douyin. A higher value indicates a louder voice for the keyword on the platform. Internet influencer accounts: Accounts on the Douyin platform, which has published video works and has experienced growth in followers, are defined as “influencer” accounts. TGI (target group index): This index reflects the strength or weakness of the target group within a specific research scope (e.g., age group, gender, interests). A TGI of 100 represents the overall level. A higher TGI indicates greater user interest in the content. Number of Participants in Related Topics: The number of users involved in discussions related to the keyword. Video Playback Volume of Related Topics: The total number of views of videos related to the keyword. Because the tools provide aggregated, platform-level data rather than individual user metadata, traditional inferential statistics could not be performed. Instead, standardized descriptive metrics such as TGI were utilized.

## 3 Results

### 3.1 Basic data summary of bariatric surgery keywords over the last 6 months

**Figure 1:**
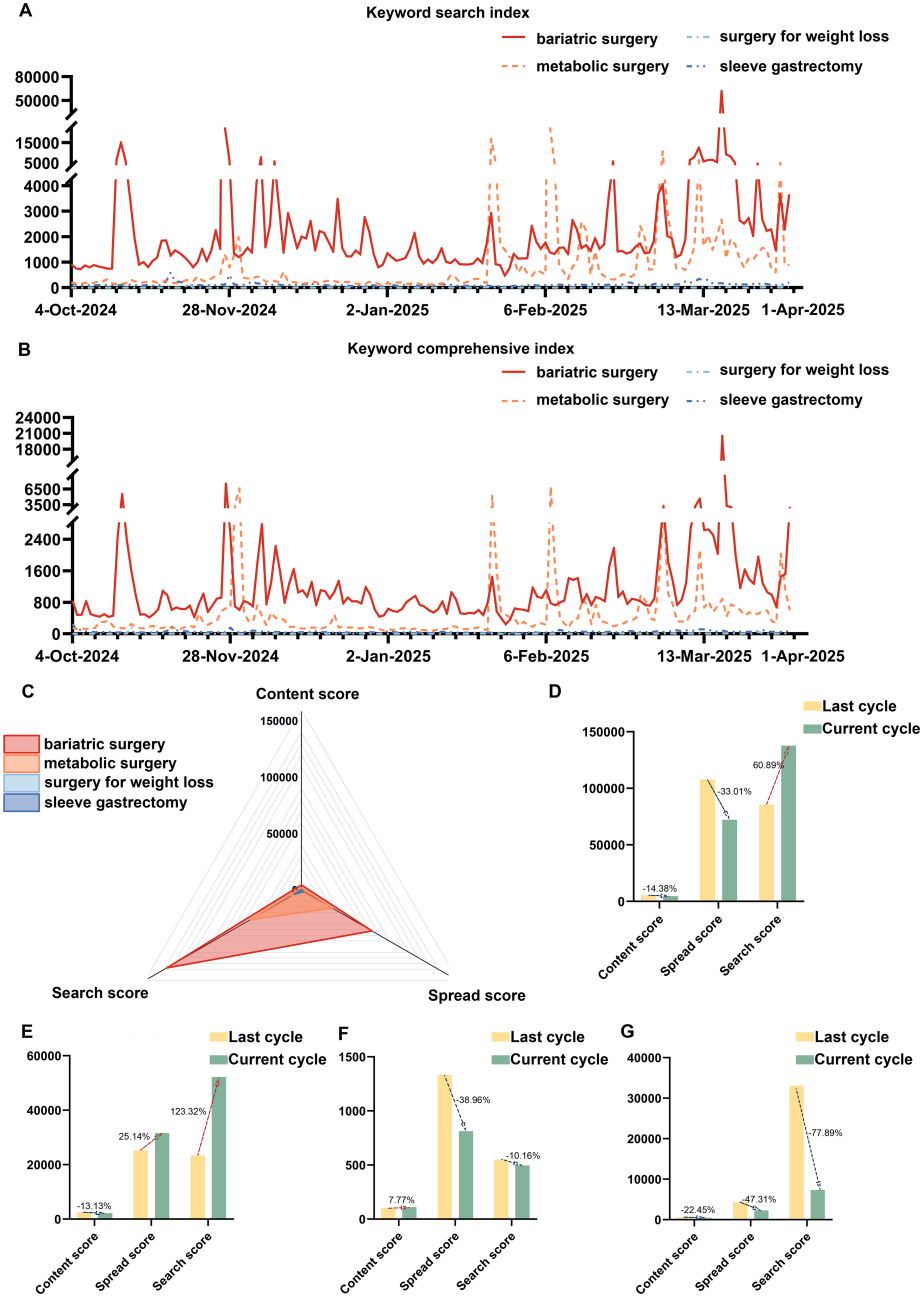
Utilize the ocean engine’s trend insight website to conduct statistics on bariatric surgery related data on the Douyin platform. **(A)** The search popularity of four keywords on Douyin. **(B)** The comprehensive popularity of the four keywords on Douyin. **(C)** The three components of the comprehensive index of the four keywords are content, dissemination, and search. **(D)** The changes in three indicators of “bariatric surgery” in the past 6 months. **(E)** The changes in three indicators of “metabolic surgery” in the past 6 months. **(F)** The changes in three indicators of “surgery for weight loss” in the past 6 months. **(G)** The changes in three indicators of “sleeve gastrectomy” in the past 6 months.

Using the “Arithmetic Index” module of Ocean Engine’s Trend Insight website, four keywords related to bariatric surgery topics, namely “bariatric surgery,” “surgery for weight loss,” “sleeve gastrectomy,” and “metabolic surgery” were added in sequence. The scope of the investigation included all users of the Douyin portal in China. Between October 4, 2024, and April 1, 2025, the search indices for “bariatric surgery” and “metabolic surgery” increased, with year-on-year growth rates of 95.02 and 97.95%, respectively, and quarter-on-quarter growth rates of 61.00 and 123.67%, respectively. The average value for the phrase “bariatric surgery” was the highest at 2547, followed by the phrase “metabolic surgery” at 964. In contrast, the search indices for “surgery for weight loss” and “sleeve gastrectomy” decreased, with average values of 7 and 133, respectively (Figure 1A). The comprehensive indices for “bariatric surgery” and “metabolic surgery” also increased, with year-on-year growth rates of 61.38 and 76.01%, respectively, and quarter-on-quarter growth rates of 7.88 and 68.55%, respectively. The average value for the phrase “bariatric surgery” was the highest at 1191, followed by the phrase “metabolic surgery” at 477. In contrast, the comprehensive indices for “surgery for weight loss” and “sleeve gastrectomy” decreased, with average values of 7 and 55, respectively (Figure 1B).

The comprehensive index was decomposed into three dimensions: a content score, which is derived from the weighted sum of the number of articles and videos related to a keyword on Douyin, reflecting the underlying voice of the keyword; a dissemination score, which is derived from the reading/viewing volume of articles/videos related to the keyword, reflecting the dissemination voice of the keyword on Douyin, note that the dissemination score is not equal to the actual reading/viewing volume; and a search score, which is derived from the weighted sum of search volumes related to the keyword, reflecting the search situation of the keyword on Douyin. The period from October 4, 2024, to April 1, 2025, is defined as the current cycle. The last cycle is the period of the same length immediately preceding the current cycle. As shown in Figure 1C, over the past 6 months, the content score was the lowest among the three dimensions for all four keywords, the dissemination score was in the middle, and the search score was the highest. The content and dissemination scores for “bariatric surgery” decreased, with a search score growth rate of 60.89% (Figure 1D). The content score for “metabolic surgery” also showed a downward trend, while the dissemination and search score growth rates were 25.14 and 123.32%, respectively (Figure 1E). In contrast, the performance of “surgery for weight loss” and “sleeve gastrectomy” was less satisfactory, with all indicators decreasing except for a slight increase in the content score for “surgery for weight loss” (Figures 1F,G).

### 3.2 Number of accounts and quality of content related to bariatric surgery

**Figure 2:**
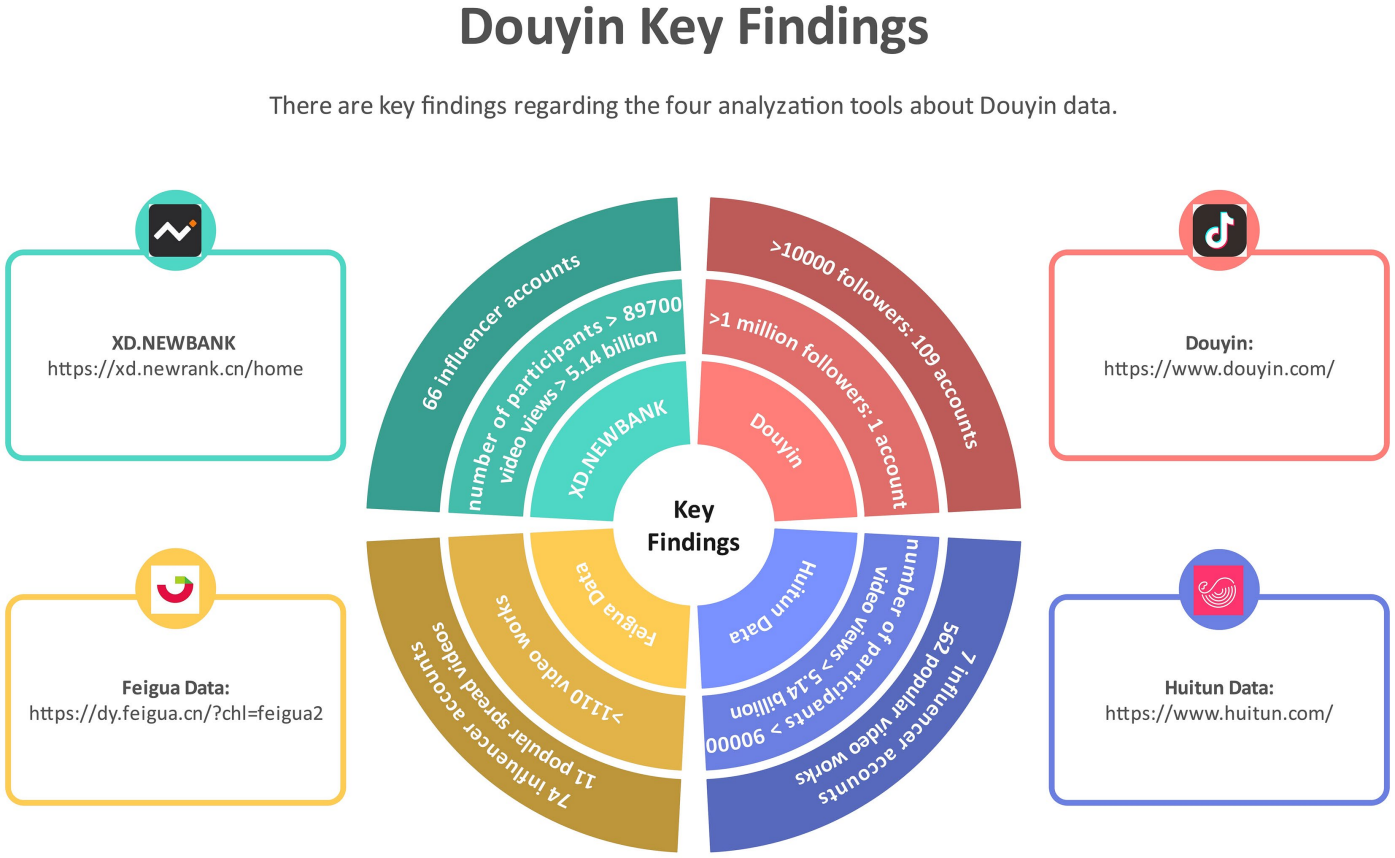
A summary of data related to bariatric surgery topics on Douyin using various analytical tools.

In the medical field in China, becoming an internet influencer has become an aspiration for an increasing number of clinical doctors, and there is a logic: to have followers have a patient base, and the more patients there are, the higher the perceived status of the doctor. This is particularly true for the emerging fields of bariatric and metabolic surgery. On the basis of the above data statistics, the keyword with the highest average search and comprehensive indices, “bariatric surgery” was selected for subsequent data analysis. Since April 1, 2025, on the Douyin platform, there has been 1 account with more than 1 million followers; 29 accounts with more than 100,000 followers, most of whom are team leaders in the field of bariatric and metabolic surgery; and 109 accounts with more than 10,000 followers. According to the data from the Huitun Data website, the number of views participating in “bariatric surgery” on the Douyin platform was 90,000, with a total video audience of 5.14 billion. The website identified 7 “influencer” accounts and 562 popular video works based on data from March 5 to April 3, 2025. According to data from the Feigua Douyin website, on the basis of data from March 5 to April 3, 2025, approximately 1,110 videos were published, with 74 “influencer” accounts identified and 11 popular videos. On the Xindou website, 66 “influencer” accounts were identified, with 89,700 people participating in the “bariatric surgery” topic and a total video playback volume of 5.14 billion, which is consistent with the statistics from the Huitun Data website (Figure 2).

### 3.3 Associated keywords of video content and viewers overview

**Figure 3:**
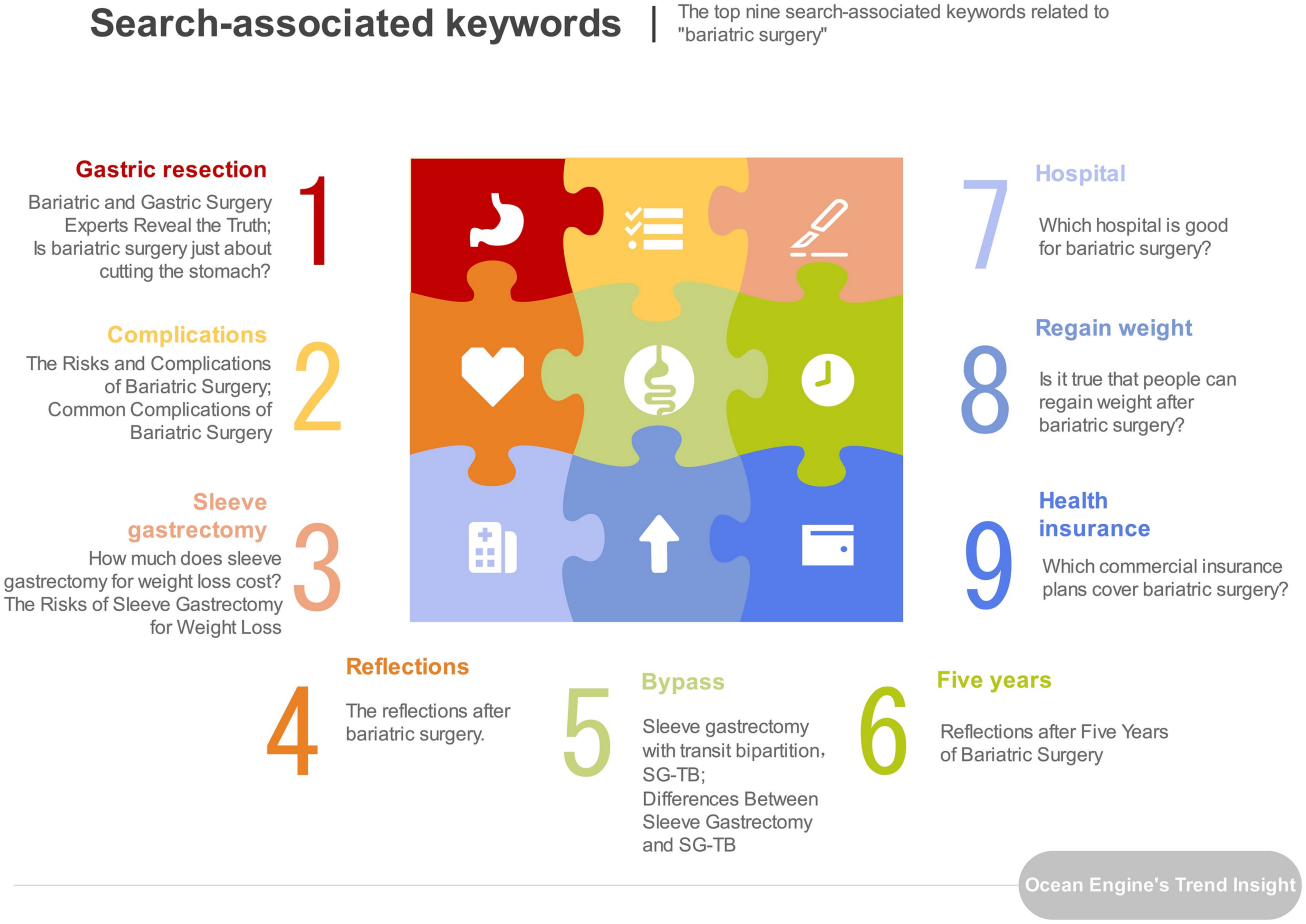
The top nine keywords related to “bariatric surgery” content on Douyin.

**Figure 4:**
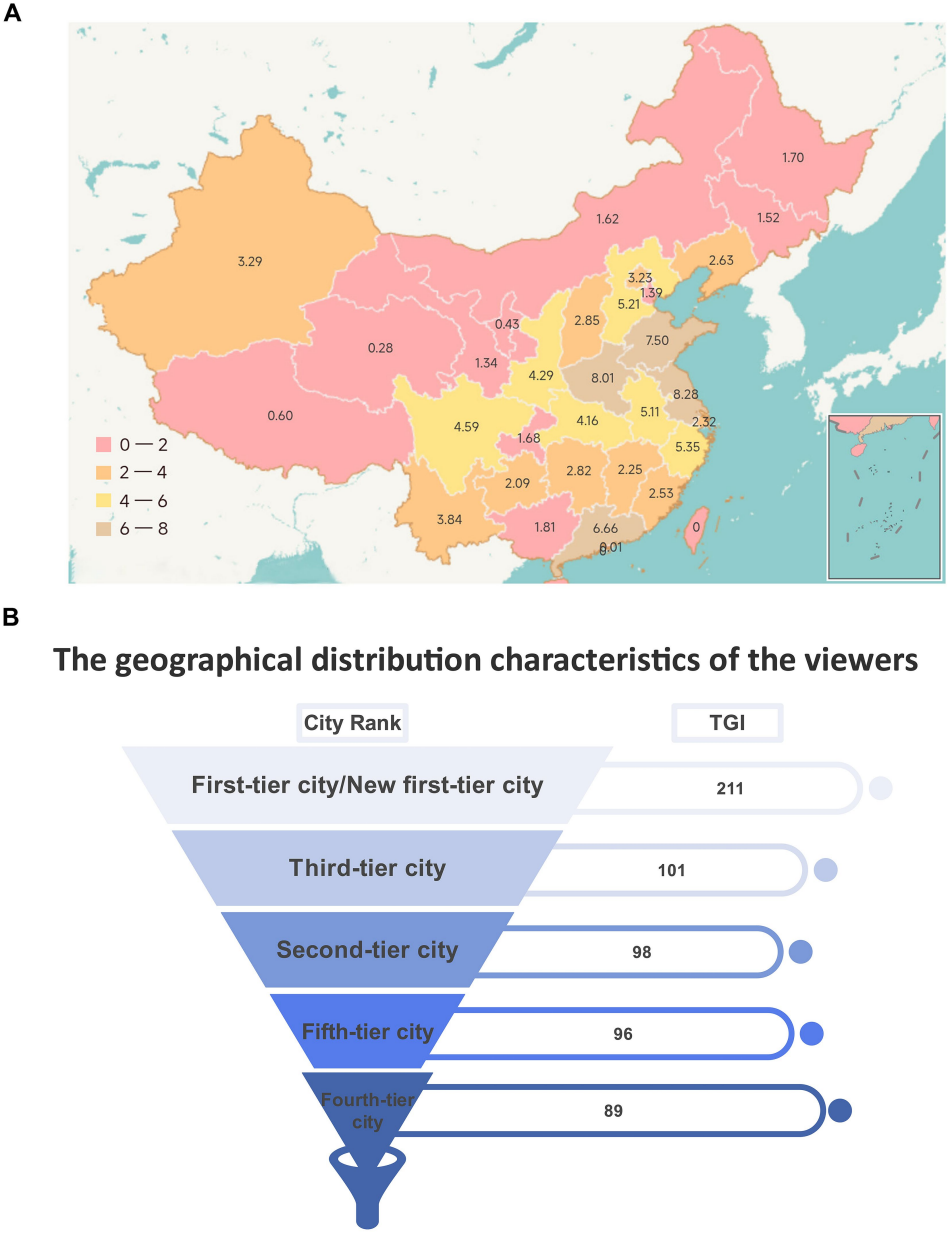
The geographical distribution characteristics of the population who have watched “bariatric surgery” videos on Douyin. **(A)** The proportion of the viewers in different provinces. **(B)** The TGI of the viewers in different levels of cities.

**Figure 5:**
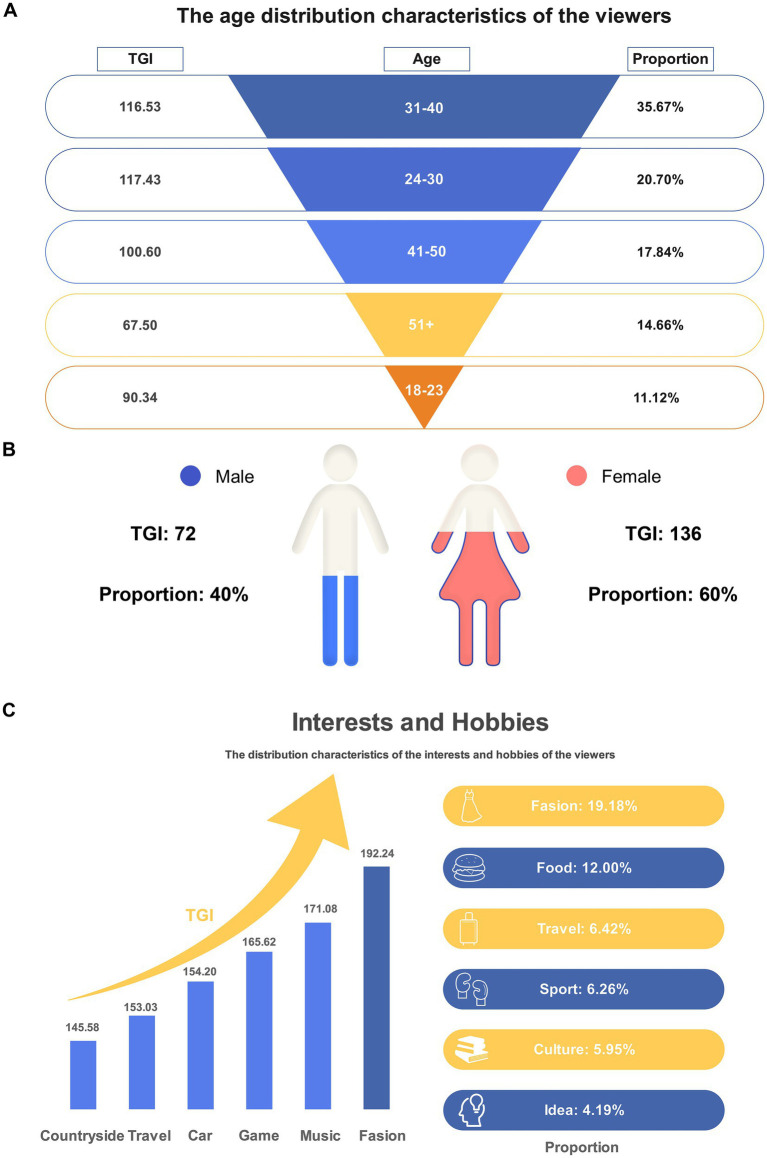
The characteristics of the viewers that watch videos related to “bariatric surgery.” **(A)** The age distribution characteristics of the viewers. **(B)** The sex distribution characteristics of the viewers. **(C)** The distribution characteristics of the interests and hobbies of the viewers.

The top nine search-associated keywords related to “bariatric surgery” on the Ocean Engine’s Trend Insight website from March 24 to March 30, 2025, were as follows: gastric reduction, complications, sleeve gastrectomy, reflections, bypass, 5 years, hospital, regain weight and health insurance (Figure 3). The higher the ranking is, the more likely users are to search for keywords together. The demographic profile of the viewers who consumed content related to “bariatric surgery” over the past 6 months was also analyzed. Content consumers are users who watched, interacted with, or otherwise engaged with content related to this topic. Analysis of the geographic distribution of people who have watched such videos revealed that Jiangsu Province had the highest proportion of viewers (Figure 4A), with the highest TGI indices in first-tier and new first-tier cities, indicating that the more developed the city is, the greater the level of attention to bariatric surgery (Figure 4B). The age distribution of viewers revealed that the 31–40 year age group had the highest proportion, whereas the 24–30 year age group had the highest TGI index (Figure 5A). The gender distribution of viewers revealed that females accounted for the highest proportion at 59%, with a TGI index of 136, compared with 72 for males (Figure 5B). The interest distribution of viewers revealed that those with an interest in fashion had the highest proportion at 19.11%, with the highest TGI index of 191.57 (Figure 5C).

## 4 Discussion

Interpreted through the lens of infodemiology—which maps the distribution and determinants of health information online—our findings highlight the pivotal role of Douyin in shaping public health perceptions. The notable increase in search indices for “bariatric surgery” and “metabolic surgery” reflects a surging public demand for medical information. However, the concurrent decrease in content and dissemination scores reveals a concerning vulnerability in the digital health ecosystem: a critical gap between high public interest and the supply of high-engagement, evidence-based content. From a theoretical perspective, when credible health information is insufficiently disseminated, the resulting digital vacuum is frequently filled by esthetically driven or scientifically unfounded content, thereby increasing the risk of misinformation. This dynamic underscores an urgent need for public health authorities to actively intervene and develop new governance frameworks. As outlined in recent models of digital transformation and the X.0 Wave Theory, building trust and resilience within these hybrid, AI-enabled health ecosystems is critical for effective post-pandemic public health communication (8).

A demographic analysis revealed a notable gender gap, with women showing greater interest in bariatric surgery content than men. Interestingly, in China, the prevalence of overweight and obesity is typically higher among men than women (9). This divergence suggests that the current public engagement with bariatric surgery online may be heavily driven by esthetic considerations and societal pressures regarding female appearance. However, alternative explanations must also be considered. Women generally exhibit higher digital health literacy and more proactive health-seeking behaviors online. This phenomenon aligns with broader global trends regarding gender disparities in obesity treatment utilization, as highlighted by recent frameworks analyzing societal adaptation to digital health transformations (10). Therefore, it is critical to shift the narrative from purely cosmetic outcomes to comprehensive metabolic health. Public health campaigns within emerging AI-enabled digital health ecosystems must adopt a gender-sensitive approach to bridge this gap, ensuring that men—who are clinically highly in need of bariatric interventions—are also effectively reached and engaged (11).

Our finding that engagement with bariatric surgery content may be heavily driven by esthetic considerations rather than evidence-based medical information aligns with recent international research on similar platforms. For instance, studies evaluating bariatric surgery videos on global platforms like TikTok and YouTube have consistently demonstrated that while patient-shared personal experiences and cosmetic outcomes garner immense viewership, the overall medical and educational quality of these videos remains suboptimal (12, 13). The proliferation of high-engagement but scientifically superficial content exacerbates the risk of health misinformation. Systematic reviews on digital health communication emphasize that social media algorithms inherently favor emotionally resonant or visually striking content, often at the expense of scientific accuracy (14). This dynamic highlights the necessity for healthcare professionals to adopt more engaging, platform-tailored communication strategies without compromising medical integrity.

Furthermore, viewed through the framework of digital health communication, the current content creation ecosystem on Douyin presents structural challenges. Currently, the generation of bariatric surgery content is driven primarily by individual physicians or medical institutions. While their efforts to enhance digital health literacy are laudable, the data reveal significant inconsistencies. Many doctors face time constraints balancing clinical duties and social media management, often leading to reliance on third-party proxy companies for content production. This non-standardized approach can compromise the educational integrity of the videos, failing to accurately convey the medical complexities of bariatric procedures. Effective digital health communication requires a dual competency: clinical accuracy and a deep understanding of platform-specific algorithmic engagement. To address this issue, it is recommended that health professionals work with experienced health content producers who understand both the concept of health and effective communication strategies. In addition, the establishment of guidelines or templates for the production of high-quality, informative and engaging content can help standardize the production process and ensure the consistent and accurate dissemination of information.

The data revealed differences between the prevalence and acceptance of bariatric surgery in different regions. In less developed areas, limited access to information, poor economic circumstances and traditional beliefs may discourage patients from undergoing bariatric surgery. In some regions, the traditional belief that “obesity is a symbol of health and well-being” remains, which is to some extent an obstacle to widespread support for bariatric surgery. Closing this gap requires targeted educational activities to address local concerns and misconceptions. An effective strategy could be to use local influencers and community leaders to disseminate accurate information on bariatric surgery. In addition, cooperation with local medical institutions to provide more convenient and affordable services may help increase the availability and acceptance of bariatric surgery in these areas.

The data presented in this paper provide valuable insights and point the way forward for future research. For example, further investigation of the specific factors affecting sex and regional disparities may provide the basis for developing more targeted interventions. Exploring the long-term impact of social media content on public attitudes and behaviors will help in the development of more effective strategies for education. With the development of social media analytics, we can continuously monitor and analyze user engagement and content effectiveness in real time and thus continuously improve our promotion efforts.

This study has several limitations. First, the observational and descriptive design precludes any causal inferences regarding public behavior. Second, the reliance on third-party commercial analytics platforms (which use proprietary, non-open-source algorithms) limits methodological transparency and external reproducibility, as these tools only provide aggregated metrics rather than user-level raw data, preventing formal inferential statistical testing. Third, our study did not include a structured, criteria-based evaluation of the video content (e.g., assessing medical accuracy or misinformation), meaning conclusions can only be drawn about engagement trends, not educational quality. Fourth, the selected six-month time window may be influenced by short-term viral events and might not reflect long-term platform dynamics. Finally, while our chosen keywords cover the primary terms used in China, potential biases remain as colloquial slang terms may have been excluded.

## 5 Conclusion

In short, social media platforms such as Douyin have great potential to influence public perceptions and attitudes toward bariatric surgery. However, the current situation also reveals a number of challenges to address to fully exploit the benefits of these platforms. Improving the quality of content, carrying out training activities for underrepresented groups, and increasing the efficiency of content creation are key steps in promoting a more accurate and comprehensive understanding of bariatric surgery among the public. By addressing these issues, we can move toward a future in which bariatric surgery is not only seen as a solution to weight loss but also recognized as an essential part of comprehensive care for people with obesity.

## Data Availability

The raw data supporting the conclusions of this article will be made available by the authors, without undue reservation.

## Footnotes

1
https://www.douyin.com/

2
https://trendinsight.oceanengine.com/

3
https://huitun.com/

4
https://feigua.cn/

5
https://dy.feigua.cn/?chl=feigua2

6
https://xd.newrank.cn/home

7
https://newrank.cn/home

## References

[1] Clapp B Ponce J Corbett J Ghanem OM Kurian M Rogers AM . American Society for Metabolic and Bariatric Surgery 2022 estimate of metabolic and bariatric procedures performed in the United States. Surg Obes Relat Dis. (2022) 20:425–31. doi: 10.1016/j.soard.2024.01.012 38448343

[2] Brown WA Liem R Al-Sabah S Anvari M Boza C Cohen RV . Metabolic bariatric surgery across the IFSO chapters: key insights on the baseline patient demographics, procedure types, and mortality from the eighth IFSO global registry report. Obes Surg. (2024) 34:1764–77. doi: 10.1007/s11695-024-07196-3, 38592648 PMC11031475

[3] Liu J. Retrospect of twenty years of development and prospect of bariatric and metabolic surgery in China. Chin J Dig Surg. (2019) 18:822–5. doi: 10.3760/cma.j.issn.1673-9752.2019.09.002

[4] Chinese Society for Metabolic and Bariatric Surgery (CSMBS), Chinese Society for Integrated Health of Metabolic and Bariatric Surgery (CSMBS IH), Chinese Obesity and Metabolic Surgery Collaborative (COMES Collaborative). Chinese obesity and metabolic surgery database: annual report 2023. Chin. J Obesity Metab Dis. (2024) 10:73–83. doi: 10.3877/cma.j.issn.2095-9605.2024.02.001

[5] Zhu C Xu X Zhang W Chen J Evans R. How health communication via Tik Tok makes a difference: a content analysis of Tik Tok accounts run by Chinese provincial health committees. Int J Environ Res Public Health. (2019) 17:192. doi: 10.3390/ijerph17010192, 31892122 PMC6981526

[6] Department of Medical Administration, National Health Commission of China. Chinese guidelines for the clinical management of obesity (2024 edition). Med J Peking Union Med Coll Hosp. (2025) 16:90–108. doi: 10.12290/xhyxzz.2024-0918

[7] Eysenbach G. Infodemiology and infoveillance: framework for an emerging set of public health informatics methods to analyze search, communication and publication behavior on the internet. J Med Internet Res. (2009) 11:e11. doi: 10.2196/jmir.1157, 19329408 PMC2762766

[8] Mattiello D. Charting public health horizons: hybrid SMEs and the X.0 wave theory in post-COVID governance. J Polit Soc. (2025) 3:2322. doi: 10.59400/jps2322

[9] Wang L Zhou B Zhao Z Yang L Zhang M Jiang Y . Body-mass index and obesity in urban and rural China: findings from consecutive nationally representative surveys during 2004–18. Lancet. (2021) 398:53–63. doi: 10.1016/s0140-6736(21)00798-4, 34217401 PMC7617101

[10] Mattiello D. Examining gender disparities in obesity clinic utilization: an analysis of sex and gender influences via the X.0 wave theory. J Polit Soc. (2025) 3:1710. doi: 10.59400/jps1710

[11] Mattiello H Cultural & gender awareness in the digital medicine/health X.0 era: a paradigmatic shift. Proceedings of the Final Colloquium, CAS in Sex- and Gender-Specific Medicine, Zurich: University of Zurich

[12] Peng G Wang C Zhang HW Xu T Di JZ. Evaluating the reliability and quality of bariatric surgery educational content on TikTok and Bilibili: a cross-sectional content analysis. Obes Surg. (2025) 35:5111–9. doi: 10.1007/s11695-025-08317-2, 41102452

[13] Batar N Kermen S Sevdin S Yıldız N Güçlü D. Assessment of the quality and reliability of information on nutrition after bariatric surgery on YouTube. Obes Surg. (2020) 30:4905–10. doi: 10.1007/s11695-020-05015-z, 32990890

[14] Suarez-Lledo V Alvarez-Galvez J. Prevalence of health misinformation on social media: systematic review. J Med Internet Res. (2021) 23:e17187. doi: 10.2196/17187, 33470931 PMC7857950

